# Breeding behaviour, visual communication and male combat of *Philothamnus occidentalis* and *Philothamnus natalensis*

**DOI:** 10.1007/s00114-025-01972-6

**Published:** 2025-02-25

**Authors:** Kirsty J. Kyle, Colleen T. Downs

**Affiliations:** Centre for Functional Biodiversity, School of Life Sciences, University of KwaZulu-Natal, Private Bag X01, Scottsville, Pietermaritzburg, 3209 South Africa

**Keywords:** Green snakes, Reproduction, Aggression display, Mating display

## Abstract

Western Natal green snakes (*Philothamnus occidentalis*) are a relatively common and widely distributed snake across the eastern half of South Africa. Little is known about their reproduction other than information that has been gleaned from others in the genus. We document several separate cases of communal breeding, as well as both aggressive and reproductive visual communication, all of which are new information for this species. This study also documents a breeding event for the eastern Natal green snake (*Philothamnus natalensis*), which shows different behaviours supporting the recent species split.

## Introduction

The western Natal green snake (*Philothamnus occidentalis*) is a relatively common species throughout its distribution range, most easily distinguishable in the field from the other *Philothamnus* species by its dark eye (Marais and Kemp 2022). Within southern Africa, it is limited to the southern and eastern parts of South Africa and Eswatini (Tolley et al. 2023). *Philothamnus occidentalis* was previously considered a subspecies of the eastern Natal green snake (*Philothamnus natalensis*); however, it was raised to a full species in 2019, based on genetic, behavioural and morphological differences (Engelbrecht et al. 2019). While habitat destruction is impacting this species, they are thought to be fairly change tolerant and persist in urban environments, and thus are considered of least concern (Tolley et al. 2023).

While little is known about their breeding, *P. occidentalis* are known to be oviparous, laying 4–15 eggs in early summer, and male combat has been observed (Branch 1998; Marais and Kemp 2022). Communal nesting events are known from the genus. *Philothamnus angolensis* is known to be a communal nester, with up to 85 eggs in a communal nest site in rotting vegetation (Branch 1998). *Philothamnus battersbyi* is also documented to lay its eggs communally, with over 100 eggs being recorded from a single site with large concentrations of over 40 individuals being seen in vegetation around the laying site, including both gravid and nongravid females and males (Spawls et al. 2004). Here, we document several cases of communal nesting as well as two different types of visual communications along with male combat for *P. occidentalis* and a further observation of a seemingly different mating strategy employed by *P. natalensis*.

## Methods

Using ad libitum event sampling methods (Altmann 1974), we collected information on *Philothamnus* spp., especially *P. occidentalis*, breeding behaviour, visual communication and male combat from 2012 to 2024 in KwaZulu-Natal and Eastern Cape provinces, South Africa.

## Results and discussion

**Fig. 1:**
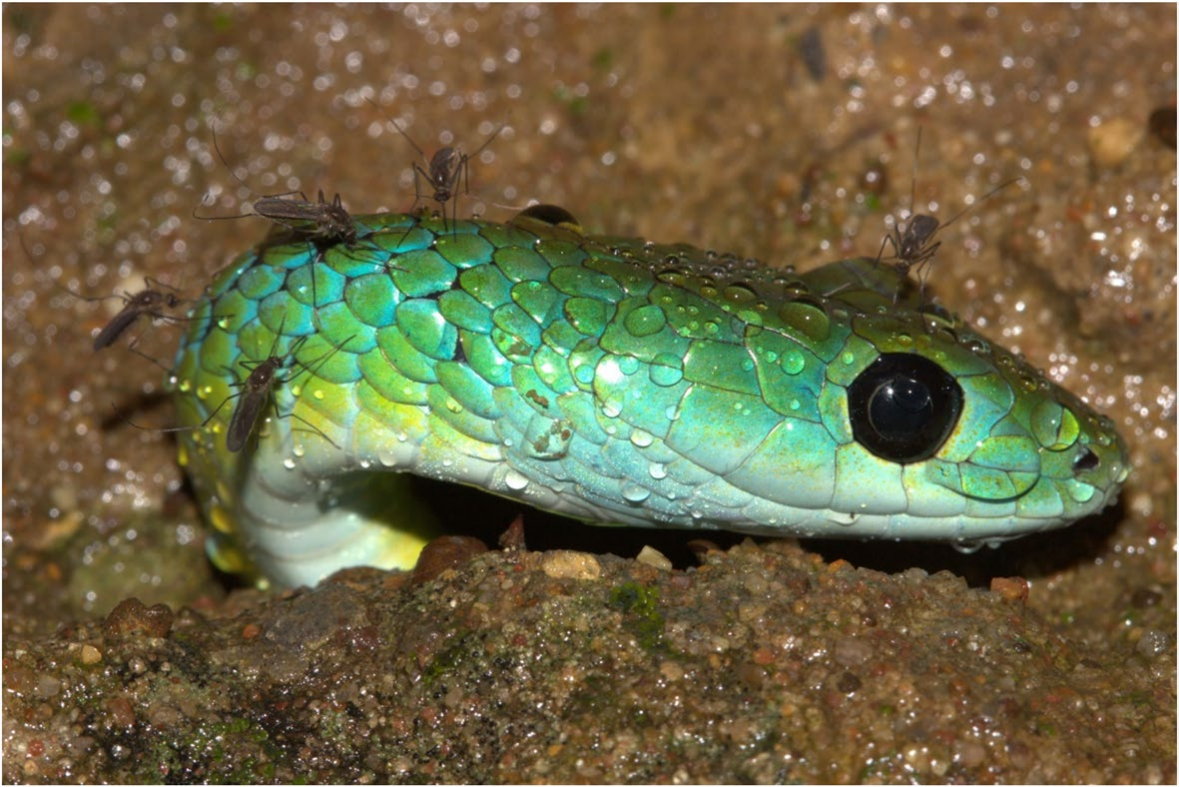
A female *Philothamnus occidentalis* laying eggs in a wall with mosquitos biting her

In November 2012, adult *P. occidentalis* were noticed congregating around a derelict stone wall in a dry riverbed on Nyamazane Game Farm in Izingolweni on the south coast of KwaZulu-Natal (− 30.758299°; 30.145307°). The snakes were going into and coming out of a hole in the wall. The site was visited frequently over several weeks, and multiple individuals were seen routinely. At least seven individuals were seen on one occasion, and upon further investigation, eggs could be seen through cracks in the stonework. On the 6th of November a large gravid female was found protruding from the hole, seemingly in the act of laying eggs, as she would not move as the others had done previously. She retracted her head as far as possible, but her head remained visible. Interestingly, multiple mosquitos were taking advantage of her immobility (Fig. 1).

On the 20th Of November 2020, whilst fishing along the riverbank of the Umzimkulu River in KwaZulu-Natal (− 30.065023°; 29.799173°), over a space of 100 m, ± 10 *P. occidentalis* were seen darting for cover in cracks in the river bank from their sunbathing positions. One was caught and found to be visibly gravid.

**Fig. 2:**
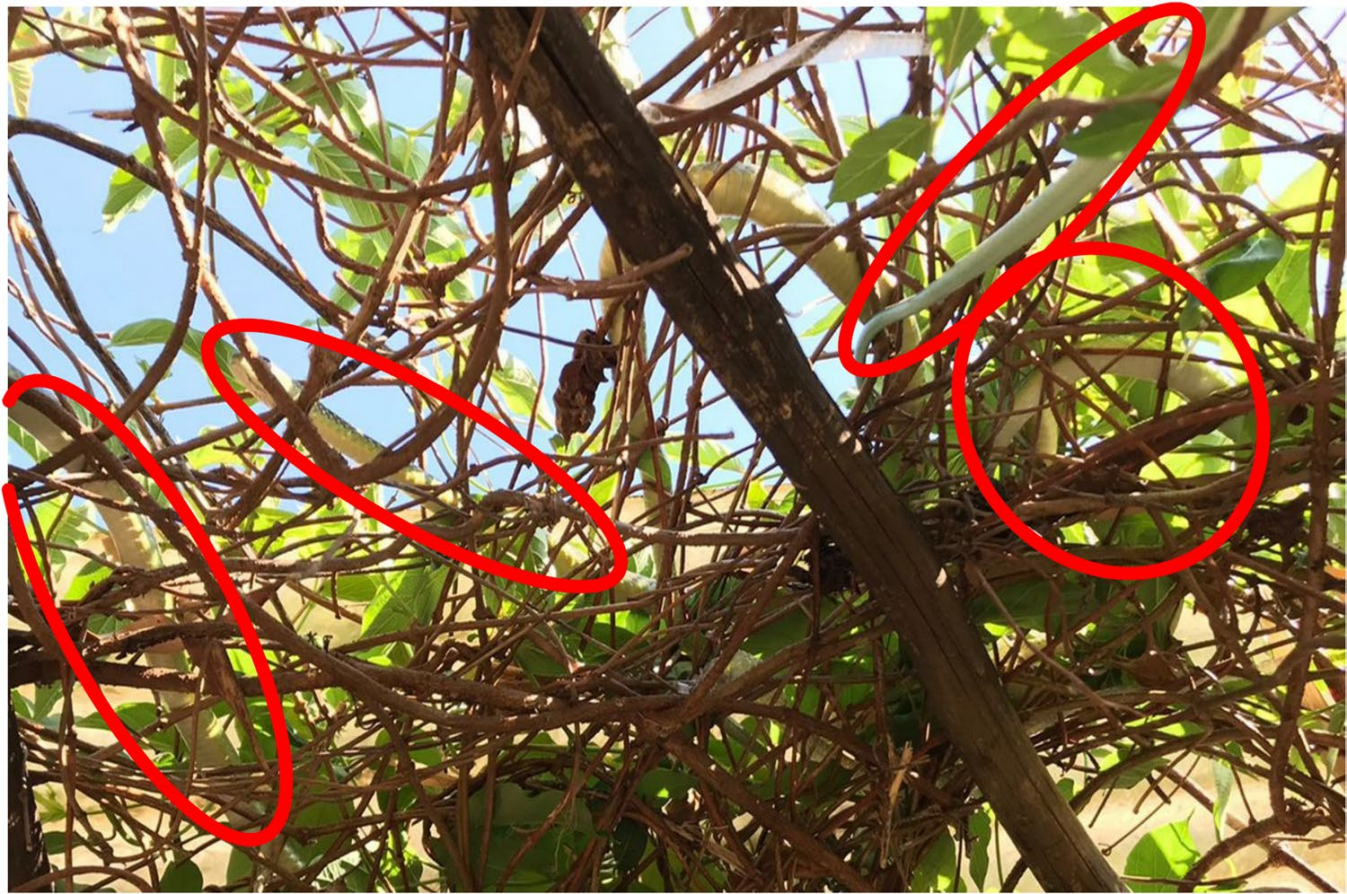
An example of *Philothamnus occidentalis* congregating and sunbathing on the vegetation at Shire Eco Lodge, Eastern Cape, South Africa

In the Eastern Cape at the Shire Eco Lodge (− 32.536728°; 27.387148°), *P. occidentalis* have been observed gathering for the last 5 years in substantial numbers from mid-September until mid-October in the garden on an arch built close to a cracked wall. The snakes gathered and sunbathed on the vegetation on the arch, frequently curling up together and on top of each other, with up to three in a coil at a time (Fig. 2). The maximum number of snakes seen on the arch at one time was 11. Some of the snakes, at least, were certainly visibly gravid. The snakes gathered like this for several weeks and then dispersed into more expected numbers in the garden for the rest of the season. There was an inundation of juveniles when the eggs hatched in summer. The adults were seen entering cracks in the wall routinely throughout the duration of their congregation there and were assumed to be laying eggs in the wall as the hatchlings emerged in this area.

On the 13th of September 2024, at Zingela Safari Camp on the Tugela River in KwaZulu-Natal (− 28.716443°; 30.062681°), a large *P. occidentalis* was seen on the top of a stone chimney on the roof of the lodge; a smaller snake was seen on the wall of the chimney; and a third, smaller snake was seen on the roof next to the chimney. Upon approach, the snake on the wall disappeared into a hole in the wall, and the other two remained where they were, out of reach. Upon observation, the smaller snake on the roof approached the chimney, after which the large individual on the top launched off his perch and rapidly chased the approaching snake, who retreated several metres. Once it had pursued the snake for a metre or two, the larger individual raised the front quarter of its body about 10 cm off the roof and started to sway its neck in a rippling, undulating fashion. The smaller snake noted this and retreated, disappearing over the arch of the roof and out of site. The larger snake then returned to the chimney. Shortly thereafter, the small snake emerged from the hole and started to climb up the wall again. The larger snake noticed this and came down from the top towards the smaller one, flicking its tongue rapidly, and when it got within 10 cm of the other snake, it started to bob its head up and down frenetically while continuing its tongue flicking and advancing on the other snake. The smaller one hardly reacted at all other than to continue on its path up the wall and into a nearby tree. The larger snake carried on interacting while the smaller snake’s head was close by, but once it had moved away, the larger snake returned to its post on the top of the chimney. A short while later, a fourth snake appeared on the scene, also a large individual, and the first one once again launched off its vantage point and pursued the intruder, this time chasing it persistently through the surrounding vegetation and, at one point cornering it against a tree where it lay on top of it for a brief while before it escaped and was pursued again until both snakes were exhausted, and the newcomer managed to escape into the bushes.

On the south coast of KwaZulu-Natal (− 30.241839°; 30.781472°) on the 9th of October 2024, a breeding congregation of *Philothamnus natalensis* was observed in the roof beams of a vehicle garage. Judging by the one’s larger size and the behaviour of the snakes, there appeared to be one female and at least six males accompanying her, with at least one successful coupling event. The entire process, from when the snakes began to gather until when they all dispersed, took ~ 30 min, suggesting that the female released a signal, perhaps a pheromone, that attracted males from the vicinity. No aggression or communication was displayed between the males; they were jostling for position and access to the female.

From these incidents, it is apparent that *P. occidentalis* uses communal nest sites, with aggregations of females happening in mid to late austral spring (October to November), with the eggs frequently laid in cracks in walls or riverbanks in close proximity to water. *Philothamnus occidentalis* appears to use at least two types of communication, one aggressive and the other mating-related. The raised, undulating movement appeared to be an intimidation and aggression display, possibly between two males to avoid combat. The aggressive attack, pursuit and combat with the fourth snake, assumed to be a larger male, perhaps would not be easily intimidated by a display and thus warranted the attack. *Philothamnus hoplogaster* have been recorded in male combat, although in that incident, the two snakes had their posterior halves tightly entwined (Marais and Midlane 2010). The close proximity, frenetic head bobbing and tongue flicking are likely a male’s mating advances on a female, as this is similar behaviour to what has been observed prior to mating in other snake species previously (pers. obs.). *Philothamnus natalensis* appear to have mass mating events with several males involved; however, there was no apparent breeding display or communication in the observed incident. It is interesting that for *P. natalensis*, multiple males were present with no aggression, while *P. occidentalis* in a potentially similar situation displayed extremely aggressive behaviour, providing further evidence of the behavioural difference between the two separate but once-clumped species and supporting this split. While both communal nesting and male combat have been recorded for the genus, this is the first record of both for this species, as well as the first documentation of visual communication between these snakes.

## Data Availability

The data belong to the University of KwaZulu-Natal. They are available from the corresponding author upon reasonable request.
